# The impact of the experimental “Hypoxic Boxing” training on the motor abilities and specialized fitness of national boxing champions: a randomized controlled trial

**DOI:** 10.3389/fphys.2025.1550659

**Published:** 2025-03-20

**Authors:** Tadeusz Ambroży, Piotr Snopkowski, Łukasz Rydzik, Andrzej Kędra, Wojciech Wąsacz

**Affiliations:** ^1^ Department of Sport Theory and Motor Skills, Institute of Sport Sciences, University of Physical Culture in Kraków, Kraków, Poland; ^2^ Polish Boxing Association for Professional Boxing, Warsaw, Poland; ^3^ Department of Sport Science, Faculty of Social Sciences, Vincent Pol University, Lublin, Poland

**Keywords:** combat sports, experimental intervention, IHT, Normobaric Hypoxia, motor fitness, specialized performancecombat sports, hypoxic chamber

## Abstract

**Introduction:**

Among theorists and practitioners, there is a consensus regarding the significant role of optimizing sports training in high-altitude conditions. This stems from the specific combination of environmental variables that determine the dynamics of changes in broadly understood training adaptation. The aim of this study was to evaluate the impact of an experimental training program, Hypoxic Boxing (under normobaric hypoxia conditions), on the development of the functional profile (motor and specialized fitness) within a national elite group of boxers.

**Methods:**

A randomized controlled trial was conducted with 20 elite-level boxers representing the national championship level (mean age: 23.9 ± 3.0 years; height: 181.3 ± 7.14 cm; body weight: 79.3 ± 8.84 kg; BMI: 24.15 ± 2.21; training experience: 10 ± 4.0 years). The participants were assigned to either the experimental group (Hypoxic Boxing - HB; n = 10) or the control group (Normoxic Boxing - NB; n = 10). Both groups followed the same 6-week training program, which included two daily training sessions (morning and afternoon). The afternoon training sessions for the HB group were conducted under normobaric hypoxic conditions in a hypoxic chamber, while the NB group trained in non-simulated normoxic conditions. The profile of changes was assessed before and after the intervention (pretest vs. posttest) by analyzing the results of selected motor ability tests from the Eurofit battery and specialized fitness using the Pawluk Boxing Test.

**Results:**

The HB group (within-group analysis) demonstrated a significant improvement in test performance for strength endurance and resistance to fatigue in the abdominal, arm, and back muscles. Specifically, the number of sit-ups increased from 27.5 ± 4.0 to 28.8 ± 3.4 (*p* = 0.007, *d*_*c*_ = 0.35), and the number of pull-ups improved from 14.9 ± 4.5 to 16.4 ± 4.6 (*p* = 0.005, *d*_*c*_ = 0.33). The intervention also led to a notable enhancement in specialized fitness, including anaerobic capacity and technical efficiency, as reflected in the number of punches delivered in 20 s (72.6 ± 9.6 to 74.3 ± 9.5, *p* = 0.008, *d*_*c*_ = 0.18), post-exercise recovery (HR 1 min: 143.3 ± 6.6 to 138.4 ± 5.8 bpm, *p* = 0.004, *d*_*c*_ = 0.79), and the multidimensional Index of Specialized Performance (4.5 ± 0.5 to 4.3 ± 0.5, *p =* 0.005*, d*_*c*_ *=* 0.40). These changes were not observed in the NB group (*p* > 0.05). Additionally, the HB group showed increased homogeneity in performance outcomes during the post-test phase. The intergroup comparison of training effects after the experiment revealed significant differences in the overall dimension of special fitness (*p* > 0.05), with a more favorable improvement observed in the HB group.

**Conclusion:**

Hypoxic Boxing demonstrates the benefits of an extended, combinatory training program compared to standard protocols. Our findings hold both scientific and practical significance, as Hypoxic Boxing appears effective in enhancing selected motor abilities and multidimensional specialized fitness. Further research is needed to better understand the potential benefits and limitations of hypoxic training for combat sports athletes.

## 1 Introduction

Boxing, also referred to as “the art of fencing with fists,” is the flagship discipline among striking combat sports, including those classified as Olympic sports (Kelestimur et al., 2004; Litwiniuk et al., 2022). It is practiced both at an amateur and professional level, with the pairing of competitors and the duration of bouts depending on the type of boxing, weight category, age group, and gender (Obmiński et al., 2011; Karpiński, 2020). On a technical level, competitors engage in standing positions (distance, mid-distance, clinch), utilizing specialized boxing locomotion techniques (e.g., boxing stance, sidesteps, leaps). Additionally, to gain an advantage over their opponent, they execute a variety of offensive actions (single or combination attacks: straight punches, hooks, and uppercuts) and defensive maneuvers (sidesteps, dodges, blocks, and covers) (Ambroży et al., 2015). Considering these variables, a skilled boxer should possess comprehensive motor fitness, a wide range of technical and tactical skills, and excellent physical conditioning to meet the demands of training and competition (Chaabène et al., 2015; Ambroży et al., 2016). A particularly crucial aspect is the synergy of these components, or in other words, the athlete's specific ability to function effectively within their discipline, defined as multifaceted specialized fitness (Wąsacz et al., 2024). The overarching goal of sports training is for the athlete to achieve optimal per-formance (Spieszny et al., 2024). This drives the pursuit of innovative training methods and the assessment of their effectiveness, aiming to optimize the broadly understood training process, as emphasized in numerous publications (Čepulėnas et al., 2011; Ouergui et al., 2014; El-Ashker, 2018; Çakmakçı et al., 2019).

Among theorists and practitioners, there is a consensus on the significant role of optimizing sports training in high-altitude conditions (Khodaee et al., 2016; Ramchandani et al., 2024). The effectiveness of altitude training depends on several key factors, including altitude level (typically 2000–4,000 m at 760–620 hPa), the duration of exposure (2–3 weeks for significant physiological adaptations), and the time frame in which the effects persist post-training (which may last from several days to a few weeks, depending on individual adaptation levels and training intensity). This is due to the specific combination of environmental variables that influence the dynamics of changes in broad-based training adaptation. The most critical and unique factor is the reduced availability of oxygen (hypoxia), which compels the athlete's body to undergo intensified adaptations to better cope with oxygen deficiency. This includes, among other things, an increase in erythropoietin production, blood volume, and oxygen-carrying capacity. Following recovery, the physiological adaptation positively impacts exercise performance (Mujika et al., 2019).

Considering the limiting criterion, namely the lack of desired access to high-altitude terrain, an alternative may lie in anthropotechnology, interpreted as the relationship between humans and technology. More specifically, this involves the use of specialized hypoxic and thermoclimatic chambers (normobaric or hypobaric) in training. These devices enable the artificial induction of various combinations of climatic conditions (control of altitude, temperature, and humidity, as well as the ability to create environmental conditions that do not naturally occur) (Pałka et al., 2023). The literature suggests an improvement in athletes' exercise performance following hypoxic training (Wilber, 2007). To enhance athlete functionality, one training model involves living at low altitudes while training at specific high-altitude conditions (Live Low/Train High - LL/TH). A combinatory training method using normobaric hypoxic devices is Intermittent Hypoxic Training (IHT), where athletes participate in sessions lasting 30–90 min under controlled hypoxic conditions while staying in normoxic conditions before and after the session (Ambroży et al., 2020). It is important to note that while the above-mentioned IHT studies refer to conditions simulated using normobaric hypoxia, other protocols may employ hypobaric conditions achieved by actual ascent to high-altitude environments (e.g., Mujika et al., 2019). The physiological response to hypoxia is harnessed to improve exercise capacity. Scientific reports on IHT indicate enhancements in the potential for prolonged submaximal intensity exercise (evidenced by increases in maximal oxygen uptake - VO_2_ max and intensity at metabolic thresholds, particularly at the second ventilatory threshold - VT_2_), which is significant in competitive sports (Dufour et al., 2006; Czuba et al., 2011). However, other studies have not observed an increase in VO_2_ max under IHT conditions, potentially due to insufficient duration of hypoxia exposure or inadequate training loads (Levine, 2002; Levine and Stray-Gundersen, 2007). Diving deeper into the topic, additional research reports that hypoxic training increases anaerobic power, which enhances the effectiveness of short-duration exercises at maximal and supramaximal intensities (Meeuwsen et al., 2001; Hendriksen and Meeuwsen, 2003; Faiss et al., 2013; Puype et al., 2013). In contrast, other studies suggest that such training does not significantly impact anaerobic performance or selected motor abilities (e.g., explosive lower-limb strength, maximum running speed) (Brocherie et al., 2015; Sanchez and Borrani, 2018).

Given the conflicting reports on the effectiveness of IHT and the ongoing quest to optimize the functional profile of boxing athletes, it is worth considering the experimental design of a training program incorporating IHT. Such an initiative would allow for an assessment of the impact of this application on variables that determine training functionality and athletic performance. There is a notable lack of studies verifying the application of IHT within the combat sports domain. To the best of our knowledge, we are the first to explore the diagnosis of motor and specialized fitness changes, personalized specifically for boxing athletes. Accordingly, the aim of this study was to evaluate the impact of an experimental training program conducted under normobaric hypoxia on the development of the functional profile (selected motor abilities and specialized fitness) in a group of elite-level, national-level boxing athletes.

## 2 Materials and methods

The project obtained approval from the Ethics Committee at the District Medical Chamber in Krakow, under the reference number No. 226/KBL/OIL/2023. In accordance with the Helsinki Declaration requirements, the participants were informed about the research objectives, methods, potential side effects, and the option to withdraw from the study at any time without providing a reason. The participants provided written con-sent to participate.

### 2.1 Study design

An experimental approach with repeated measurements and a randomized controlled trial design was employed. The testing procedure was conducted before and after a 6-week intervention period. For the experimental group (HB - Hypoxic Boxing), the intervention was integrated into their regular training program, with modifications to their afternoon training sessions, which were conducted under normobaric conditions in a hypoxic chamber. The control group (NB - Normoxic Boxing) followed the same training program; however, all sessions were conducted outside the chamber, exclusively under normoxic conditions interpreted as those naturally occurring in the Krakow region of Poland. It was a deliberate experimental measure aimed at reliably comparing the effect of training under simulated high-altitude conditions with that of traditional training methods, which are commonly carried out under normoxic conditions.

### 2.2 Participants characteristics

The study involved a purposively selected group of 20 Polish male athletes who were actively training in boxing at a national championship level. The participants' average age was 23.9 ± 3.0 years, with a mean height of 181.3 ± 7.14 cm, body weight of 79.3 ± 8.84 kg, BMI of 24.2 ± 2.2, and an average training experience of 10 ± 4.0 years. The sample size was calculated using G*Power v 3.1.9.6 (effect size f = 0.65, α = 0.05) with anactual power of 80%. Initially, 24 competitive players were recruited. Four of them were excluded from the study due to exclusion criteria (history of injuries or health status). The inclusion criteria for the study were as follows: national championship-level boxer, current medical clearance, no history of severe injuries, positive medical recommendation, abstinence from supplementation (during the study period) and doping, and active participation in competitions. Exclusion criteria included the violation of any of the above conditions, as well as current injuries or conditions that may affect participation in training or studies, lack of consent from the athlete for participation in the study, athletes with no experience in participation in sports competitions, spending more than 48 h at an altitude exceeding 2,000 m above sea level within 6 months prior to the study. All participants were active competitors in championship-level events, including international, national, and local competitions, with some achieving notable sporting successes. The study was conducted during the preparatory phase of the training cycle. The athletes were not on restrictive diets and, during the intervention period, did not participate in competitions or sparring sessions at competition-level intensity. Information on chronological age, training experience, activity level, and competitive history was gathered using a diagnostic survey method, implemented through direct interviews with the athletes and their coaching staff.

Selection of research groups (procedure): Based on purposeful subgroups defined by weight categories, the participants were divided into two groups. At this stage, the allocation was random: one participant from each weight category was randomly assigned to one of the two study groups: HB (n = 10) or NB (n = 10). The study included athletes from the following weight categories: Lightweight (n = 5), Middleweight (n = 7), Heavyweight (n = 8). Each category was evenly distributed between the experimental and control groups. Each participant was assigned a unique identification number, and group placement was determined through a random number generator. The three-stage selection process (Stages 1 and 2 purposeful, Stage 3 random) allowed for the creation of groups with similar body mass, height, and BMI values. No significant differences were found between groups (Table 1).

**TABLE 1:** Statistical characteristics of basic somatic traits in the studied groups of athletes (HB vs. NB).

Variables	Group HB (n = 10); $\tilde{x}$ ± sd	Group NB (n = 10); $\tilde{x}$ ± sd	p-value
Body height [cm]	181.8 ± 4.88	180.3 ± 4.19	0.471
Body weight [kg]	80.4 ± 8.02	78.5 ± 8.22	0.607
BMI [kg/m^2^]	24.3 ± 2.11	24.2 ± 2.44	0.923

HB, hypoxic boxing; NB, normoxic boxing; $\tilde{x}$ , arithmetic mean; sd, standard deviation; *p*, level of significance.

### 2.3 Characteristics of the experimental intervention

**FIGURE 1:**
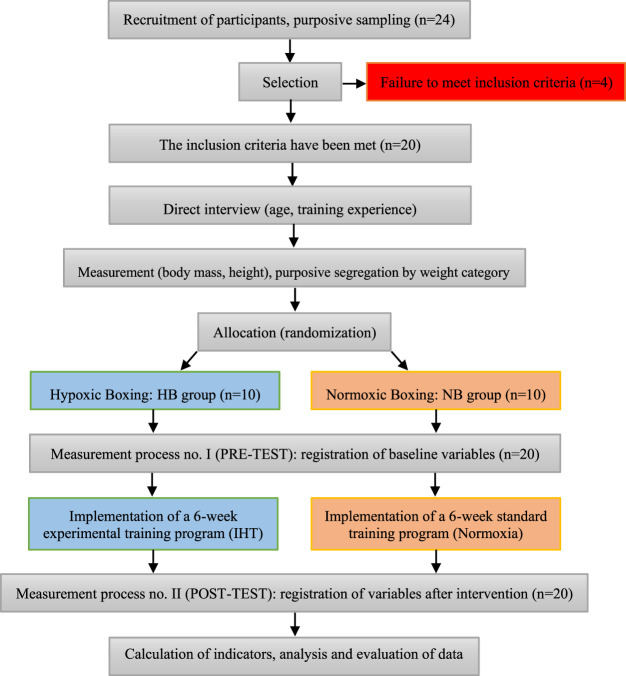
Flowchart of the research intervention.

The participants in both groups (HB and NB) followed the same training program, consisting of 4 days per week with 2 training sessions per day, over a period of 6 weeks. The control group (NB) performed all training sessions in normoxic conditions outside the hypoxic chamber to prevent any potential placebo effect. The duration of 6 weeks was selected based on previous studies investigating hypoxic training, which indicate that this period allows for measurable physiological adaptations, such as increased erythropoiesis and enhanced anaerobic performance (Wilber, 2007; Mujika et al., 2019). Additionally, a 6-week training cycle aligns with typical preparatory phases in elite boxing training schedules. In the morning, they performed a 60-min technical-tactical boxing training session, which included low and moderate-intensity exercises (up to 50% of maximum load). In the afternoon, they completed a 60-min session aimed at developing and enhancing endurance and motor abilities. The total weekly training time was 480 min. The difference in the applied intervention was that the boxers in the HB group carried out their afternoon training session under normobaric hypoxia. The hypoxic chamber (Wichary Technologies, Żory, Poland) measured 5 m × 5 m × 3 m and allowed for simultaneous training of up to 5 athletes. The CO_2_ levels inside the chamber were maintained below 0.5%. IHT was applied in a normobaric hypoxic chamber (Wichary Technologie, Żory, Poland) at a simulated altitude of 4,000 m (FiO_2_ = 12.9%), with a temperature of 21°C–22°C and air humidity between 40% and 45%. The athletes in the NB group completed their training cycle in Kraków (natural climate), exclusively in normoxic conditions (230 m above sea level). To assess the profile of changes induced by the intervention, two measurement points were used. The first measurement was taken before the start of the program (baseline diagnosis of variables: pre-test). The second measurement was taken after the completion of the 6-week training period (effectiveness assessment: post-test). The final tests were conducted 24 h after the completion of the last training session. The chosen time interval allowed for adequate athlete recovery, eliminating the influence of immediate fatigue and transient effects, while simultaneously enabling the evaluation of lasting training adaptations (Mujika et al., 2019). The impact of the experimental training was evaluated within the HB group (intra-group) and compared to the NB group (inter-group), which served as a reference point for comparison. A flow diagram of the research intervention is presented in Figure 1.

### 2.4 Characteristics of the applied training program

The first training session in the morning (9:00–10:00) focused on the technical and tactical aspects of boxing, with the specifics outlined in Table 2. The second training session in the afternoon (18:00–19:00) consisted of two phases (see Table 3). The first phase (a 3-week application period) was based on various functional training guidelines using bodyweight resistance exercises (Ambroży, 2008) in a circuit training format (Ambroży, 2007). The second phase (the subsequent 3-week intervention) was focused on developing and enhancing endurance abilities, utilizing the airbike Airdyne AD7 (Schwinn, Chicago, United States).

**TABLE 2:** Structure of the technical-tactical training unit in a morning session.

Preliminary part (10 min)	Main part (40 min)	Final part (10 min)
7 min general segment: light jog; shaping exercises according to the gymnastic routine	First 2 weeks of the training period: 15 min column-based technical exercises: single punches or combinations (straight-hooks-uppercuts) performed together with steps (forward-backward-left-right)	7 min flexibility and mobility exercises
3 min specialized segment: shadow boxing	25 min technical exercises in pairs: one trainee delivers punches alternately single or in combinations (straight-hooks-uppercuts) together with steps (forward-backward-left-right), while the other trainee performs alternating defenses (block-parry-slip-lean-back-step-backside-step-ducking).	3 min relaxation exercises, calming the body
	Next 4 weeks of the training period: Same training content performed from the opposite boxing stance to the usual one	

**TABLE 3:** Structure of the motor and effort training unit in an afternoon session.

Preliminary part (10 min)	Main part (40 min)	Final part (10 min)
7 min general segment: light jog; shaping exercises according to the gymnastic routine	First 3 weeks of the training period (strength endurance): 5 min dynamic stretching exercises 5 min free work with a jump rope 30 min station-based circuit: Resistance – body weight Number of exercises per circuit – 6 Number of circuit repetitions – 4 Exercise duration – 30 s Tempo – fast Passive rest between circuits – 180 s No breaks between exercises within a circuit 1st station: Squat jump 2nd station: Push-ups 3rd station: Russian twists 4th station: Lunges 5th station: Plank 6th station: Mountain climbers	6 min flexibility and mobility exercises
3 min specialized segment: shadow boxing or coordination ladder exercises		4 min relaxation exercises, calming the body
	Subsequent 3 weeks of the training period (effort abilities): 5 min dynamic stretching exercises 5 min free work with a jump rope 30 min interval effort (airbike): Interval program 20/50: 8 rounds, 20 s work at maximum subjective intensity/interspersed with 50 s recovery interval performed at four times lower intensity, program performed twice	

The key priorities of the program were its structure (exercise content, sequence, number of sets, and repetitions), which encompassed aspects related to technical-tactical skills, muscular fitness, and physical endurance. The exercises were designed and implemented with the aim of optimizing a broad hybrid of specialized fitness, a crucial area for the effective performance of an athlete in their specific sport discipline (Wąsacz, 2023).

### 2.5 Measurements of motor and specialized fitness

Each time before the measurements, both groups participated in a standard 15-min warm-up session consisting of exercises to prepare the body for physical effort. All tests were performed in normobaric conditions. Exercises were conducted in accordance with the principle of formative exercises and involved static and dynamic movements of the arm, trunk, abdomen, back, and legs. The examiner demonstrated each test according to the procedure, then provided instructions and clarifications. Effective recovery breaks (at least 15 min) were observed between trials.

Motor fitness, in the context of selected motor abilities, was assessed using standardized population tests from the EUROFIT battery (European Physical Fitness Test) (Grabowski and Szopa, 1989) and general physical fitness tests (Talaga, 2004). The selection of motor tests was based on years of athlete-coach practice (choosing those trials that are subjectively the most useful for the physical demands of elite boxers). Additionally, the EUROFIT protocol includes the bent-arm hang test, which assesses the potential for force generation in an isometric dimension, a priority plane in grappling combat sports (Wąsacz and Pociecha, 2021; Wąsacz et al., 2022; Wąsacz et al., 2023). The pull-up test (Talaga, 2004) was applied as a more appropriate measure of strength fitness in relation to the specificity of boxing. The testing procedure included the following trials:

1. Standing long jump (explosive power): the subject stands with the feet slightly apart in front of the starting line, bends the knees, and moves the arms backward at the same time, and then he or she performs the arm swing and jumps as far as they can; the landing occurs on both feet while maintaining the upright position; the test is performed twice. The longest of the two jumps measured to the closest mark left by the participant'sheel is recorded, with an accuracy of 1 cm. A tape measure, a hard surface, and two gymnastic mattresses connected lengthwise are used.
2. Static handgrip strength measurement: The participant, standing with feet slightly apart, held the dynamometer firmly in the fingers, with the arm positioned along the torso, ensuring the hand did not touch the body. The participant performed a brief maximal grip, while the other arm rested alongside the body. The best result of two maximal static strength tests of the dominant hand (HGSmax) was recorded using a dynamometer (MG 4800, Charder, Taichung, Taiwan) with an accuracy of 1 kg. The rest interval between tests was 5 min.
3. Sit-ups (strength endurance of the abdominal muscles): The participant lay on a mat with feet 30 cm apart and knees bent at a 90-degree angle. Hands were clasped behind the neck, and a partner held the participant's feet. At the start signal, the participant sat up, touched the knees with elbows, and returned to the starting position. The duration was 30 s, and the result was the number of repetitions completed.
4. Pull-ups on the bar (arm strength): evaluation of the strength of the shoulder girdle based on the number of repetitions: the subject catches the bar using a pronated grip and hangs; at the signal, the subject bends arms in elbows and pulls the body up so high that the chin is above the bar and then, without rest, returns to a simple hanging; the exercise is repeated as many times as possible without rest; the result is the number of complete pull-ups (chin over the bar).

**FIGURE 2:**
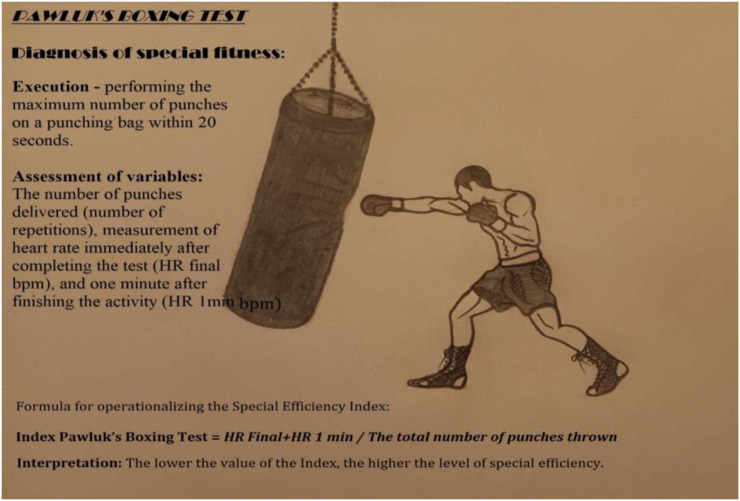
Graphical diagram of the Pawluk's Boxing Test execution. Source: Authors' own elaboration.

To diagnose specialized fitness, the Pawluk Boxing Test (Fidziński, 1982) was used. While high-altitude training is primarily associated with improvements in aerobic endurance, the present study focused on anaerobic capacity and sport-specific performance. Therefore, a traditional aerobic test such as the 20 m shuttle run was not included. Instead, the Pawluk Boxing Test was selected as a more relevant measure of short-duration high-intensity effort. The posttest assessments were conducted 24 h after the last training session to minimize the impact of acute fatigue and ensure reliable performance measurements. This is a specific and specialized research tool for assessing and monitoring the level of this variable in boxing activity (Adamczyk et al., 2013; Szot et al., 2017). According to the test protocol, the test involved performing the maximum number of punches on a punching bag within 20 s. The participant assumed a fighting distance, directly and frontally in front of the punching bag, adopting a boxing stance with a guard on their dominant side. Upon the “box” command, the participant began the trial, executing any punches (straight, hook, or uppercut). The procedure required performing the punches with maximum speed under supramaximal effort conditions. The “stop” command signified the end of the trial. The evaluation focused on: the number of correctly landed punches. This was assessed retrospectively based on a video recording played in slow motion. The heart rate (HR bpm) was also measured immediately after the test (HR final), and 1 minute after completing the test (HR 1min) (see Figure 2). Following Sterkowicz's method developed for judo (Sterkowicz, 1995), the Special Endurance Indexes of the Pawluk Test were calculated according to the following formula:

$$Index\;Pawluk\prime s\;Boxing\;Test=\frac{HR\;\;\;final\;\left(bpm\right)+HR\;1\;\min.\;\left(bpm\right)}{The\;total\;number\;of\;punches\;thrown}$$

Where:

HR final - heart rate recorded immediately after completion of the test.

HR 1min - heart rate recorded 1 min after the completion of the test.

The total number of punches thrown - The number of punches delivered in 20 s.

The index reflects the level of a boxer's special endurance, which signifies the effective collaboration of the body's exertional capabilities, overall fitness, and technical skills. The interpretation of the test result is inversely proportional–the higher the level of special endurance, the lower the value of the index (Ambroży et al., 2021; Chwała et al., 2023; Wąsacz, 2023; Wąsacz et al., 2024).

### 2.6 Statistical analysis

In the analysis of the research results, basic statistical methods were used, including the calculation of the arithmetic mean, median, standard deviation, minimum and maximum values, and the coefficient of variation. To assess the significance of differences between groups, the independent samples t-test or the non-parametric Mann–Whitney U test was used. To evaluate the significance of within-group changes (differences in the progression of a given group), the paired samples t-test or the non-parametric Wilcoxon signed-rank test for paired observations was applied. Furthermore, the effectsize was calculated using Cohen's d index (d = 0.20, weak effect; d = 0.50, moderate effect; d = 0.80, strong effect). The choice of tests was preceded by checking the normality of variable distributions using the Shapiro–Wilk test, which confirmed normality for some variables (NB: standing long jump p = 0.221, pull-ups p = 0.334, final HR p = 0.341, HR 1 min p = 0.061, index p = 0.622. HB: grip strength p = 0.212, sit-ups p = 0.324, number of punches p = 0.287, final HR p = 0.075, HR 1 min p = 0.201, index p = 0.124), while indicating a significant deviation from normality for others (NB: grip strength p = 0.043, sit-ups p = 0.012, number of punches p = 0.016. HB: standing long jump p = 0.031, pull-ups p = 0.023. The degree of intragroup diversity was as-sessed by interpreting the coefficient of variation values according to the classification: CV < 25% indicates low variability; 25%–45% indicates moderate variability, 45%–100% indi-cates high variability; >100% indicates very high variability (Cohen, 2013). The collected data were analyzed using Statistica software, version 13.3 (Statsoft, Krakow, Poland). The threshold for statistical significance was set at *p* < 0.05.

## 3 Results

### 3.1 Results of the assessment of the profile of changes in selected motor abilities

The test effects of the studied groups and their level of intergroup (HB vs NB) and intragroup differentiation are presented in Table 4.

**TABLE 4:** The statistical characterization of the profile of selected motor abilities and their variability (intergroup and intragroup) in the studied groups (HB vs. NB) of boxers (n = 20).

Measurement	Group HB (n = 10)			Group NB (n = 10)			d_1_	*p*_*1*_	*d*_*c1*_
	$\tilde{x}$	sd	CV(%)	$\tilde{x}$	sd	CV(%)			
Standing long jump - explosive power [cm]									
I pre-test	234.6	13.3	5.7	226.2	12.5	5.5	8.2	0.162	0.65*
II post-test	235.4	13.4	5.7	231.4	9.7	4.2	4.0	0.455	0.35
d_2_	0.8	1.6	202.4	5.2	3.0	57.2	−4.4	**0.002**	1.91*
*p*_*2*_	0.153			**0.005**			-	-	-
*d*_*c2*_	0.06			0.47**			-	-	-
Static handgrip strength measurement [kG]									
I pre-test	54.3	6.1	11.2	54.0	6.5	12.1	0.3	0.894	0.05
II post-test	54.7	6.2	11.3	54.4	5.7	10.4	0.3	0.938	0.05
d_2_	0.38	1.2	302.6	0.37	1.5	402.9	0.01	0.762	0.01
*p*_*2*_	0.150			0.479			-	-	-
*d*_*c2*_	0.07			0.07			-	-	-
Sit-ups - strength endurance of the abdominal muscles [Number of repetitions]									
I pre-test	27.5	4.0	14.4	26.1	2.9	10.9	1.4	0.375	0.41**
II post-test	28.8	3.4	11.8	26.7	2.8	10.6	2.1	0.150	0.68*
d_2_	1.3	1.2	89.2	0.6	1.0	161.0	0.7	0.151	0.64**
*p*_*2*_	**0.007**			0.081			-	-	-
*d*_*c2*_	0.35**			0.21			-	-	-
Pull-ups on the bar - arm strength [Number of repetitions]									
I pre-test	14.9	4.5	30.1	16.5	4.4	26.8	−1.6	0.433	0.36**
II post-test	16.4	4.6	28.1	17.8	4.3	24.4	−1.4	0.493	0.31
d_2_	1.5	1.5	100.6	1.3	1.2	89.2	0.2	0.521	0.15
*p*_*2*_	**0.005**			0.074			-	-	-
*d*_*c2*_	0.33			0.30			-	-	-

HB, hypoxic boxing; NB, normoxic boxing; $\tilde{x}$ , arithmetic mean; sd, standard deviation; CV%, coefficient of variation; I, first measurement period (pre-test); II, second measurement period (post-test).

d, difference between means (delta); *p*, level of significance; level of significance statistically significant values are shown in bold (p < 0.05); *d*_*C*_, effect size expressed using Cohen's *d* coefficient (intergroup); * strong effect; ** moderate effect; _*1*_, data concerning inter-group variability; _*2*_, data concerning intra-group variability.

With respect to the baseline assessment (first measurement–pretest), the groups exhibited a similar level of selected components of motor fitness, as measured by population tests, with no significant differentiation observed (p > 0.05). However, significant effect sizes were noted for the results of the long jump and sit-ups from a lying position, both favoring the HB group, with the effect sizes being strong and moderate, respectively. An opposite trend was observed for pull-ups on a bar, where the NB group performed better, with a moderate effect size.

After 6 weeks of applying the experimental stimulus (second measurement–posttest), intergroup comparisons did not reveal significant differences in the levels of the test variables (p > 0.05). However, in order to isolate the initial level of variables in the groups, the analysis of the mean difference comparison showed a significant variation for the standing long jump (p = 0.002), where a more favorable progress was observed in the NB group. The HB group demonstrated better abdominal muscle endurance in the sit-ups test, with a strong effect size noted.

Comparative analysis of intragroup progression revealed significant differentiation for improvements in the efficiency of sit-ups (p = 0.007) and pull-ups (p = 0.005) within the HB group. For the NB group, such an effect was observed only for explosive strength, as measured by the standing long jump (p = 0.005).

The variability coefficients indicate that internal differentiation of the analyzed test variables within both groups (HB vs NB) was very low (CV = 4–24%). Regarding the efficiency of strength endurance in arm and back muscles, as assessed by the pull-up test, moderate internal differentiation bordering on very low was observed in both measurement points (CV = 24.4–30.1%).

### 3.2 Results of the assessment of the profile of changes in specific fitness variables

Table 5 presents the profile of changes in the components of special fitness as-sessed using the Pawluk Boxing Test, as well as the degree of intergroup and intragroup differentiation within the studied groups of boxers (HB vs NB).

**TABLE 5:** The statistical characterization of variables related to specific fitness and their variability (intergroup and intragroup) in the studied groups (HB vs NB) of boxers (n = 20).

Measurement	Group HB (n = 10)			Group NB (n = 10)			d_1_	*p*_*1*_	*d*_*c1*_
	$\tilde{x}$	sd	CV(%)	$\tilde{x}$	sd	CV(%)			
The total number of punches thrown [number of repetitions]									
I pre-test	72.6	9.6	13.2	71.9	9.8	13.6	0.7	0.762	0.07
II post-test	74.3	9.5	12.7	72.4	10.5	14.6	1.9	0.496	0.19
d_2_	1.7	1.0	55.8	0.5	1.2	235.7	1.2	**0.034**	1.09*
*p*_*2*_	**0.008**			0.236			-	-	-
*d*_*c2*_	0.18			0.05			-	-	-
HR final [bpm]									
I pre-test	178.9	5.9	3.3	180.2	5.8	3.2	−1.3	0.624	0.22
II post-test	179.5	5.9	3.3	181.3	7.4	4.1	−1.8	0.554	0.27
d_2_	0.6	4.2	699.2	1.1	3.0	275.9	−0.5	0.764	0.14
*p*_*2*_	0.662			0.281			-	-	-
*d*_*c2*_	0.10			0.17			-	-	-
HR 1 min [bpm]									
I pre-test	143.3	6.6	4.6	142.2	7.7	5.5	1.1	0.736	0.15
II post-test	138.4	5.8	4.2	141.4	7.3	5.2	−3.0	0.323	0.46**
d_2_	−4.9	4.0	−81.9	−0.8	1.9	−241.5	−4.1	**0.009**	1.39*
*p*_*2*_	**0.004**			0.223			-	-	-
*d*_*c2*_	0.79*			0.11			-	-	-
Index Pawluk's Boxing Test									
I pre-test	4.5	0.5	11.2	4.5	0.5	12.0	0.0	0.829	0.00
II post-test	4.3	0.5	10.9	4.5	0.6	12.6	−0.2	0.411	0.36**
d_2_	−0.2	0.1	−84.9	0.0	0.1	−435.8	−0.2	**0.010**	2.00*
*p*_*2*_	**0.005**			0.487			-	-	-
*d*_*c2*_	0.40**			0.00			-	-	-

HB, hypoxic boxing; NB, normoxic boxing; $\tilde{x}$ , arithmetic mean; sd, standard deviation; CV%, coefficient of variation; I, first measurement period (pre-test); II, second measurement period (post-test).

d, difference between means (delta); *p*, level of significance; level of significance statistically significant values are shown in bold (p < 0.05); *d*_*C*_, effect size expressed using Cohen's *d* coefficient; * strong effect; ** moderate effect; _*1*_, data concerning inter-group variability; _*2*_, data concerning intra-group variability.

The baseline assessment (pretest) revealed a similar intergroup level for all test variables (p > 0.05).

In the HB group, following 6 weeks of experimental training (pretest vs posttest), improved results were observed across all aspects of special fitness. The observed intergroup variation for the averaged results (HB vs NB groups) was characterized by a lack of significant differences (p > 0.05). However, in the analysis of mean differences, significant differences were noted for the number of punches (p = 0.034), post-exercise recovery–HR 1 min (p = 0.009), and the multidimensional Special Performance Index (p = 0.010), with a more favorable training effect observed in the HB group.

The comparative analysis (within-group) of test efficiency gains showed significant variation for quantitative technical performance–number of punches delivered (p = 0.008), post-exercise recovery–HR 1 min (p = 0.004), and the multidimensional Special Performance Index (p = 0.005) in the HB group, where significantly more favorable results were observed. Such cause-and-effect relationships were not found in the NB group (p > 0.05).

The variability coefficients demonstrated that the internal variability of the analyzed test variables was very low in both groups (CV = 3.2–14.6%). The averaged CV% values from the two measurement sessions indicate greater homogeneity in the HB group, with progressive improvement in homogeneity during each subsequent measurement in the experimental procedure (average test variability index for the HB group: pretest = 8.08%; posttest = 7.78% vs. the NB group: pretest = 8.58%; posttest = 9.13%).

## 4 Discussion

The aim of this study was to investigate the impact of a 6-week targeted training program, “Hypoxic Boxing,” designed to optimize selected aspects of motor abilities and broadly defined special fitness—key determinants of performance outcomes in this sport activity (Chwała et al., 2023). The experimental intervention incorporated intermittent simulated high-altitude conditions (LL/TH + IHT) combined with resistance, endurance, and boxing-specific exercises. According to theorists and discussions within the practical sports community, training under hypoxic conditions is gaining significance in the context of optimizing training processes, especially for endurance sports (Czuba et al., 2011), but also for specific areas of combat sports (Arabaci, 2015), including boxing (Ambroży et al., 2020). A thorough analysis of the relevant literature identified a research gap concerning the evaluation of IHT interven-tions' effectiveness in the boxing environment on general and special fitness profiles. This study aimed to address this scientific and practical gap to further develop the dis-cipline. Our findings demonstrated that this approach was effective for the HB (ex-perimental) group, leading to improvements in some motor abilities and, essentially, all verified variables that constitute the profile of special fitness, which is crucial for combat sports (Szot et al., 2017).

The analysis of intragroup differentiation revealed significant progressions in the strength endurance of the abdominal (p = 0.007), arm, and back muscles (p = 0.005), as assessed through the sit-ups and pull-ups tests. Posttest results indicated an average improvement of 1.3 repetitions in sit-ups and 1.5 repetitions in pull-ups, indicating a moderately significant improvement (d_c_ = 0.35 and 0.33). This suggests enhanced muscle fatigue resistance and an associated improvement in the body's tolerance to acidification, which can be critical during prolonged sessions of intermittent high-intensity efforts commonly observed in boxing (Slimani et al., 2017). This improvement is also significant from a technical perspective, as nearly every technical action in boxing relies on the efficient muscular and energetic function of these areas (Sharkey and Gaskill, 2013). Motor abilities related to generating maximum force in short bursts (2–10 s), assessed through hand dynamometry and the standing long jump, also showed improvement, although the changes were not statistically significant (p > 0.05). In contrast, Hagiwara et al., in their case study of two international-level fencers, reported an increase in explosive power after 3 weeks of sprint training under hypoxic conditions (Hagiwara et al., 2023). The differing outcomes might be attributed to methodological differences, as their training content specifically targeted the improvement of this variable. Interestingly, in the NB (control) group, a significant increase (p = 0.005) was observed for explosive strength, as assessed by the standing long jump. Participants in this group improved their posttest results by an average of 5.2 cm (moderately significant improvement d_c_ = 0.47). For other variables within this group, no significant improvement was noted.

General motor tests do not always correlate directly with sports performance (Ambroży et al., 2021). Therefore, it is highly appropriate to use specific research tools to diagnose and monitor the unique hybrid of an athlete's abilities required for effective performance in their discipline (Wąsacz et al., 2024). It is crucial that these tools are designed based on the technical-tactical aspects of the sport and simulate the conditions of real competitive confrontations (Wąsacz, 2023). In this study, a trend of overall improvement was also observed for multidimensional special fitness, as assessed by the Pawluk Boxing Test. The HB group demonstrated significant intragroup progress in the number of punches delivered in 20 s (p = 0.008), with an average posttest improvement of 1.7 punches. This indicates enhanced ability to deliver punches at full speed and suggests improved anaerobic performance (Zatoń and Jastrzębska, 2020). Similar findings were reported by Ran Wang et al., who observed improvements in anaerobic capacity among amateur female boxers following a short-term Live Low-Exercise High training period (21 days) (Wang et al., 2015). Likewise, Ambroży et al. found a similar trend of improvement in anaerobic performance among elite national-level boxers after a 6-week intervention (Ambroży et al., 2020). Significantly better results were also recorded for post-exercise recovery during the first minute (HR 1 min) (p = 0.004), with an average posttest reduction of 4.9 bpm. This indicates strong progression (d_c_ = 0.79). This improvement is particularly relevant for recovery between rounds during a bout, as well as during tournaments where athletes may need to compete multiple times in 1 day. As a result of these progressions, a marked improvement (p = 0.005; d_c_ = 0.40)) was observed in the operationalized indicator of broad special fitness (Index). This effect was not seen in the NB group (p > 0.05), confirming the more effective and comprehensive training adaptation of the HB group subjected to intermittent hypoxic training. These results highlight the potential of such innovative training stimuli, utilizing advanced anthropotechnics like hypoxic chambers. When analyzing the global results (motor and special fitness), a functional improvement trend can be noted for high-intensity efforts lasting 20–60 s. Qualitatively, this appears related to the improved efficiency of the glycolytic system (anaerobic glycolysis) (Sharkey and Gaskill, 2013). However, no similar improvements were observed in the HB group for short-duration efforts of 2–5 s (maximum isometric static strength in handgrip dynamometry or explosive strength in the standing long jump). This suggests that the participants' phosphagen system (ATP-PCr) did not significantly respond to the experimental intervention. Based on these observations, it can also be qualitatively inferred (Grabowski, 2022) that appropriately applying diverse training stimuli during sensitive periods (Szopa et al., 1996) is crucial for effective periodization of the training process (e.g., a macrocycle—a long-term training plan for preparation for an Olympic form or high-level competition).

A comparison between groups with a randomized control trial did not reveal the significant nature of these changes (p > 0.05). The HB group demonstrated more favorable final test outcomes, except for the pull-up test, where the NB group excelled. In order to isolate the baseline level (pretest) for each variable, an analysis of the training effects was conducted, revealing significant differentiation in the number of strikes (p = 0.034; dc = 1.09), post-exercise recovery (p = 0.009; dc = 1.39), and the special performance index (p = 0.010; dc = 2.00), with a stronger, more favorable training effect observed in the HB group. In the NB group, a similar trend was noted for the standing long jump (p = 0.002; d = 1.91). This confirms the high effectiveness of Hypoxic Boxing in the broad optimization of the training process. Future explorations should consider the proportions of exercise content, their intensity, training period, and exposure under IHT conditions. Arabaci, in his research, reported improvements in anaerobic power, 1-RM strength, and aerobic capacity in elite freestyle wrestlers after 8 weeks of intermittent hypoxic training (Arabaci, 2015). It is recommended that future interventions incorporate longer periods of the discussed stimulus to verify the potential for generating even stronger training adaptations (Škarabot et al., 2020) with more pronounced changes.

### 4.1 Limitations of the study

At this stage, the sample size can be considered a limitation of the presented study, although the participants were elite athletes in their field. Additionally, the specific profile of participants (boxers) may limit the generalizability of the results to other populations of athletes. Another limitation is the inclusion of different weight categories, as physiological demands may differ between lightweight and heavyweight athletes. To capture the multifaceted context of the problem, future studies should consider extending the intervention duration, increasing the number of participants, including female athletes, and involving representatives of other combat sports. Such measures would help confirm the findings presented in this study and provide broader insights into the effects of the training protocols examined.

## 5 Conclusion

Our findings hold scientific and cognitive significance in understanding the causal relationships between the Hypoxic Boxing training program and the dependent motor and special fitness profiles of boxing athletes. The proposed intervention in the HB group appears effective in improving selected motor abilities and overall special fitness. Despite the absence of significant changes in test outcomes measuring the potential to generate maximal force over short periods (hand dynamometry, standing long jump), the implemented program, augmented with intermittent hypoxic training (IHT), led to improved test performance in muscular endurance and fatigue resistance of the ab-dominal, arm, and back muscles (sit-ups, pull-ups) within the HB group. The experi-mental stimulus applied to the HB group significantly enhanced special fitness com-ponents, including anaerobic capacity, technical efficiency (number of punches in 20 s), post-exercise recovery (HR 1 min), and, ultimately, the multidimensional Index of Special Fitness. These improvements were not observed in the NB group. Furthermore, the progressive homogeneity of results in the HB group across subsequent stages of the experiment highlights the beneficial global impact of the program on the HB cohort. This approach underscores the advantages of adopting an extended, com-bined training program compared to standard protocols. It also emphasizes the neces-sity for ongoing exploration of innovative training interventions to optimize the func-tional profiles of athletes in this field to their maximum potential.

### 5.1 Practical implications

The 6-week Hypoxic Boxing training program can be considered an effective practical approach for shaping and enhancing the motor and special fitness profiles of elite-level boxers. The results have valuable practical implications for coaches and athletes in this sport. Hypoxic Boxing is recommended for inclusion in training practices and its evaluation in other athletic categories, including different competitive levels, age groups, and female athletes. Moreover, the potential exists to adapt the modified training program using the combined IHT method to other combat sports disciplines, where a strong motor fitness base and multidimensional special fitness are essential and determinative of athletic success.

## Data Availability

The raw data supporting the conclusions of this article will be made available by the authors, without undue reservation.
